# Effects of Attending Extracurricular Lessons and Cram School on Independent Mobility in Japanese Children

**DOI:** 10.3389/fpsyg.2022.888718

**Published:** 2022-06-13

**Authors:** Yasuo Kojima

**Affiliations:** School of Psychology, Chukyo University, Nagoya, Japan

**Keywords:** independent mobility, children, extracurricular lesson, cram school, online survey, Japan

## Abstract

Independent mobility and its related factors were examined among Japanese elementary school children. Based on the responses of 1,824 mothers with elementary school-aged children, the effects of demographic variables such as children’s grade, gender, and birth order as well as regional characteristics, neighborhood environment, distance to and means of getting to school, children’s use of bicycles, keys and cell phones, and the number of weekdays spent attending lessons or cram schools were explored. Factor analysis revealed that independent mobility comprised activities in public places, including outings to supermarkets, and traveling by bus and train as well as activities in the school district such as visiting friends’ homes and parks. Hierarchical multiple regression analyses that comprised five steps were conducted in which the number of days of attending lessons and cram school was entered in the final step. There was a strong gender effect and grade effect for outings to public places and activities in the school district. Concerns about traffic accidents and security were associated with lower independent mobility. With regard to activities in the school district, proximity to school, use of bicycles, and possession of house keys had a positive effect. It is noteworthy that the effect of the number of days spent attending lessons or cram school was observed even after the other variables were entered, thus resulting in a negative effect. It is recommended that further comparative studies involving other Asian countries be conducted to evaluate the effects of extracurricular activities.

## Introduction

In comparison to other closely related mammals, human infants have significantly inferior physical functions at birth (especially posture maintenance and mobility) and grow slowly. Accordingly, they require a caregiver, who is generally a parent, to provide extensive care and nurturing over a lengthy period. However, it is also known that there are significant cultural differences in parenting practices and developmental expectations, and that there is cultural diversity in what children experience and are allowed to do by their parents in their daily lives. In recent years, based on the consideration that much of the previous findings in the field of developmental research were largely derived from biased data collected in WEIRD (Western, educated, industrialized, rich, and democratic) countries (Henrich et al., 2010), there has been a broad trend to collect data from a variety of countries and to reconsider child development in a relative perspective. Regarding the topic of this study, children’s independent mobility, more data have been accumulated in Western countries (Sharmin and Kamruzzaman, 2017; Marzi et al., 2018), but recently there have been gradually more data published in non-Western countries (e.g., Lam and Loo, 2014; Tyagi and Raheja, 2021). This study further extends these trends by addressing independent mobility of children in Japanese sample.

As children’s mobility gradually increases, their parents tend to watch them even more carefully, partly because of the increased risk of accidents and injuries. Various reports have noted that infants suffer the majority of unintentional accidents at home in Japan (e.g., Ministry of Health Labour and Welfare, 2021). Furthermore, it is increasingly difficult for parents to spend time away from their children when they are outdoors because of the high risk of traffic accidents and crime. When children are unable to move around on their own, it is often necessary to limit their movements by employing devices such as baby carriers and strollers. Even after children can walk on their own, their parents should continue to use these devices or hold their hand in order to restrict their movements. When parents and children spend a significant amount of time away from each other, whether inside or outside the home, it is imperative that another watches them. While babysitters are often employed in developed countries, in Japan, relatives and in particular, grandparents take care of children at home (Benesse Institute for Educational Research, 2016). In addition, children often attend daycare centers in developed countries when there are no adults at home.

When children begin elementary school, they start spending an increasing amount of time engaged in a range of activities and consequent less time with their parents. In many countries, it is compulsory for children to be accompanied by their parents or other adults on their way to and from school for fear of traffic accidents and/or crimes such as being kidnapped. However, in Japan, parents do not generally accompany their children to and from elementary school. Rather, in urban areas, children of various grades who live near one another commonly gather and go to school together. However, depending on the grade, classes end at different times and thus, it may be difficult for the entire group to go home together. Moreover, parents and other adults do not usually fetch the children. Those in the same grade who live in the same neighborhood may gather and go home together or even go home alone. Once children have started elementary school, their opportunities to meet up with friends to play or attend lessons increase.

Children’s independent mobility refers to going out alone without an adult or being given permission to do so by their parents (Hillman et al., 1990). It has been known that independent mobility improves with age, and numerous studies been conducted on children older than 8 years (e.g., Prezza et al., 2005; Oliver et al., 2011; Schoeppe et al., 2015b). The extent to which independent mobility is accepted depends on a variety of factors, including societal and cultural values. Parents and their children negotiate in accordance with those values. Moore (1986) classified children’s outdoor activities into three categories: habitual range, which encompasses readily accessible neighborhoods near their home; frequented range, which includes places further away from their home that although not always accessible, are in free time; occasional range, which constitutes those places that are occasionally accessible to children and are beyond walking distance. Moore (1986) added that children gradually become involved in the local community as their range of activities increases, thus enhancing their independence and confidence.

Research has shown that children’s independent mobility has a number of positive effects on child development. Carver et al. (2014) as well as Pacilli et al. (2013) revealed that children with higher independent mobility had more enhanced physical and mental health, which may have been related to engaging in more outdoor physical activities and less time being sedentary (Stone et al., 2014). Independent mobility is also known to have positive psychosocial effects on children, possibly because they interact with friends and others more during outdoor activities (Freeman and Quigg, 2009; Schoeppe et al., 2013). Furthermore, several studies have revealed the effects of independent mobility on children’s cognitive development. Children with more independent mobility draw more detailed maps of their immediate surroundings (Holt et al., 2008), possibly because the more experience children acquire exploring different areas on their own, the more knowledge of their surroundings they accumulate (Villanueva et al., 2012). Furthermore, children who have more independent mobility experience a greater sense of belonging to their own neighborhood and a more enhanced sense of trust in society (Rissotto and Tonucci, 2002; Prezza and Pacilli, 2007). Despite the many benefits associated with independent mobility, children’s outdoor activities have been drastically reduced since the 1990s in many countries throughout the world (Karsten, 2005; Fyhri et al., 2011; Schoeppe et al., 2015a; Shaw et al., 2015; Badland et al., 2016; Scheiner et al., 2019). Data have also indicated that increasing urbanization has resulted in fewer places to play (Karsten, 2005; Little and Wyver, 2008) and greater volumes of traffic, thus increasing the risk of accidents (Lopes et al., 2014; Janssen et al., 2016; Smith et al., 2019). In addition, an increasing number of parents restrict their children from going out alone because they are concerned that their children may be involved in crimes such as kidnapping, which is often referred to as stranger danger (Prezza et al., 2005; Janssen et al., 2016; Bennetts et al., 2017). In contrast, areas with a high sense of community and social cohesion (Prezza et al., 2005; Alparone and Pacilli, 2012; Schoeppe et al., 2015a) as well as well-designed cities, which possess large parks, close proximity to public facilities, and pedestrian and bicycle paths have been linked to children’s independent mobility (Prezza et al., 2005; Chaudhurya et al., 2019). Recently, it has been suggested that because younger children use cell phones, parents find it easier not to be with their children and they may be less hesitant to allow their children to leave their home (Brockman et al., 2011; Fyhri et al., 2011). However, the results of the impact of cell phones on independent mobility vary among countries, with some studies finding no association (Rooney, 2008; Shaw et al., 2015).

One may ask how children spend their time after school. Recently, much attention has been paid to organized activities. In the United Kingdom, an increasing number of elementary school children are afforded opportunities to participate in sports, music, dance and other classes, and leisure activities that are managed by schools, communities, and private organizations. These activities are considered to be similar to after-school activities such as sports, including swimming lessons, and music in Japan (*naraigoto*). Furthermore, there is an increasing number of children in other developed countries who participate in these activities (Casper and Smith, 2004; Skar and Krogh, 2009; Hofferth and Sandberg, 2001; Nordbakke, 2019). It has been suggested that this has contributed to the accelerated decline in independent mobility (Burdette and Whitaker, 2005; King and Howard, 2014). Accordingly, children’s activities are being restricted to organized, adult-supervised places to which parents spend much of their time driving their children. Karsten (2005) referred to these children as the backseat generation and highlighted the many children being transported to various locations in the backseat of cars. Similarly, some researchers have described this type of lifestyle for children as insularization (Zeiher, 2001), thus implying that adults drive children from island to island. A similar phenomenon in Japan is that children are enrolled in lessons at a younger age. Benesse Institute for Educational Research (2016), one of the largest organizations in the educational industry in Japan, found that approximately 30% of 3-year-olds and more than 80% of 6-year-olds attend various kinds of lessons. Nishikawa et al. (2003) revealed that 35.5% of first- to third-graders attended lessons three or more days a week. Furthermore, Omiya et al. (2011) found that while fourth- to sixth-grade students in Tokyo played a considerable deal outside on days when they did not attend lessons, the content of their play indoors mainly comprised video games on days they had lessons. Ishihama et al. (2020), in a survey on children’s after-school activities among children between 8 and 12 years old, found that most children acknowledged they played with digital devices. Moreover, those who reported playing with digital devices most often attended cram schools and did not play outside much.

Although organized after-school activities, including lessons, have attracted attention in many countries, the impact of such activities on children’s development has not been examined sufficiently. Moreover, most of the research on children’s independent mobility has been conducted in Europe, United States, and Oceania (Sharmin and Kamruzzaman, 2017; Marzi et al., 2018). However, with the exceptions of Amemiya (2012) and Shimada et al. (2010), children’s mobility has not been well researched in Japan and other Asian countries. Participation in these organized activities has been demonstrated to reduce children’s free activities considerably because they are often dependent on parental transportation, which, in turn, results in lower independent mobility (Holloway and Pimlott-Wilson, 2014). Accordingly, the present study was conducted to clarify the effects of lessons on Japanese elementary school-aged children’s independent mobility.

## Materials and Methods

### Participants and Procedure

A market research company in Japan surveyed 2,100 respondents. The respondents came from nuclear families that comprised parents and children, with at least one child in elementary school. Where a family had two or more children in elementary school, the mother was required to complete the survey in relation to her eldest child. Accordingly, data were collected from 350 grade one to grade six children. There were an equal number of boys and girls. When conducting a study through a market research company, the existence of dishonest respondents or survey satisficing (Oppenheimer et al., 2009), is a problem in terms of the reliability of the data. Although there are many ways to identify those participants (Maniaci and Rogge, 2014), in this study, in accordance with Miura and Kobayashi (2015), several filler items were included, for example an instruction to answer 4 for a particular question was included. Those who did not follow such instructions were considered not to have read the question properly and were excluded from the analysis. In addition, data of twins were excluded because it was not possible to determine which child the mother had considered when answering each question. Furthermore, those who consistently could not match their child’s birth month with the school year as well as those who answered each question by employing the same value on the scale were excluded from the analysis. The final analysis comprised data from 1,824 respondents (921 boys and 903 girls). The average number of responses for each grade was 304, ranging from 285 in grade 1 to 321 in grade 3. The results further revealed that 400 were only children, 939 first-born children with younger siblings, and 485 s or subsequent children. The average age of the mothers and fathers was 39.12 and 41.31 years, respectively. The ages of the former ranged between 23 and 49 years and the latter between 23 and 69 years. Most parents were university graduates (33.5% mother and 41.8% father), followed by high school graduates (24.3% mother and 25.4% father). While 45.5% of the mothers earned less than two million yen, 34.6% did not earn an income. On the contrary, 31.3% of the fathers earned between four and six million yen, 19.8% earned between six and eight million yen, and 18.6% between two and four million yen. While the majority of mothers (44.0%) were housewives, 33.9% were employed part-time. Most of the fathers (81.7%) were employed as general workers. Detailed demographic description was also shown in Table 1.

**TABLE 1:** Demographic description of the sample.

Variables	Category	Percentage	Variables	Category	Percentage	Variables	Category	Percentage
**Children**			**Mother**			**Father**		
Gender	Male	50.5	Education	Junior high	1.4	Education	Junior high	2.7
	Female	49.5		High	24.3		High	25.4
Grade^1^	1	15.6		Technical school	15.7		Technical school	16.2
	2	17.3		Junior college	21.1		Junior college	4.8
	3	17.6		University	33.5		University	41.8
	4	16.5		Graduate school	3.7		Graduate school	8.6
	5	16.8		Others	0.2		Others	0.5
	6	16.2	Annual income (Japanese yen)	None	34.6	Annual income (Japanese yen)	None	0.3
Birth order	Only child	21.9		− 2,000,000	45.5		− 2,000,000	3.3
	First-born with younger sib.	51.5		− 4,000,000	9.3		− 4,000,000	18.6
	Second or subsequent	26.6		− 6,000,000	3.2		− 6,000,000	31.3
				− 8,000,000	1.3		− 8,000,000	19.8
				− 10,000,000	0.4		− 10,000,000	8.8
				− 15,000,000	0.2		− 15,000,000	4.2
				15,000,000 −	0.3		15,000,000 −	1.2
				DK	5.3		DK	12.3
			Employment	Employed full-time	17.6	Employment	Employed full-time	83.0
				Employed part-time	33.9		Employed part-time	1.5
				Others	4.6		Others	14.6
				None	44.0		None	0.9

*^1^In Japan, children enter elementary school in the first April after their sixth birthday.*

### Measures

#### Independent Mobility

In accordance with studies conducted in Japan (Amemiya, 2012; Kojima, 2020), we focused on the following seven activities related to going out that have been confirmed to increase progressively as children advance through the grades and do so unaccompanied by an adult: (1) home from school, (2) a friend’s house, (3) a park, (4) a supermarket or convenience store, (5) an extracurricular lesson or cram school, (6) a shopping center, and (7) using public transportation such as bus or train to go out. Mothers were asked to answer questions related to activities by selecting the most appropriate answer from the following five alternatives: “always allow my child to do this on their own,” “usually allow my child to do this on their own,” “do not allow my child to do this on their own very often,” “never allow my child to do this on their own,” and “never engages in this behavior.”

#### Area of Residence

The respondents were required to indicate whether they resided in an urban, suburban, or rural area. While 35.4% of the participants lived in urban areas, 56.1% lived in suburban areas and 8.6% in rural areas.

#### Residence

The respondents were asked whether they lived in a detached house or an apartment complex, and if in the latter, on which floor they lived. While the first to fourth floors of apartment complexes were classified as low-rise, the fifth to ninth floors and tenth and higher floors were categorized as medium-rise and high-rise, respectively. The results revealed that 65.1% of the respondents lived in a detached house, 24.3% in a low-rise apartment complex, 8.2% in a middle-rise apartment complex, 2.1% in a high-rise apartment complex, and 0.3% in other residences.

#### Distance From Home to School

The respondents were asked to specify the distance their children traveled to school. The results revealed 22.3, 35.7, 29.3, and 12.7% traveled less than 500 m, between 500 m and 1 km, between 1 and 2 km, and more than 2 km, respectively.

#### Means of Getting to School

The respondents were asked to indicate how their child traveled to school: “parents or grandparents,” “school bus,” “in a group accompanied by an adult,” “in a group unaccompanied by an adult,” “school alone,” and “other,” not specified. The results were classified in accordance with whether an adult was present or not present and whether the child went to school in a group or alone. The results showed that 10.3% of the children went to school with an adult, 51.4% with a group, 33.4% alone, and 4.9% with others.

#### Neighborhood Environments

To assess the perception of neighborhood environments, we administered a questionnaire based on the Japanese version of the environmental module of the IPAQ (Inoue et al., 2009). It is available online at http://www.tmu-ph.ac/pdf/ipaq.pdf. After deleting various items that were deemed that parents with children at elementary school would be unable to answer and adding an item, specifically availability of banks, post offices, hospitals, and other public facilities in the neighborhood, a scale of 11 items was created and administered. The respondents were asked to select one of the following options: *not at all true* (1), *not somewhat true* (2), *somewhat true* (3), and *very true* (4). Regarding this scale, a confirmatory factor analysis was conducted after an exploratory factor analysis, since some items were added or subtracted from the original scale.

#### Using a Bicycle and Carrying Keys and Cell Phone

In accordance with previous studies, the respondents were asked to indicate which of the following options were related to their children’s independent mobility: whether they used a bicycle, whether they went out with a house key, whether they owned their own cell phone, and whether they left home with their cell phone. The results revealed that 17.2, 31.6, 16.0, and 35.5% of the respondents’ children used a bicycle “often,” “sometimes,” “not often,” and “not at all or were unable to ride a bicycle”, respectively. Furthermore, 43.3% went out with a house key. While 6.7% had cell phones but never left home with them, 27.9% went out with them sometimes, and 65.5% did not own a cell phone.

#### Frequency of Lessons and/or Cram School

The respondents were also asked whether their children had lessons or cram school on each day of the week and if so, what kind of lessons they attended. The results revealed that while 596 children did not have lessons, 400 attended lessons on 1 day, 367 on 2 days, and 461 on three or more days.

### Ethical Considerations

The author obtained approval from the Ethical Review Committee of the author’s institution prior to conducting this study.

Materials and analysis code for this study are available by emailing the corresponding author.

## Results

### Factor Analysis of Independent Mobility

Exploratory factor analysis (maximum likelihood method, promax rotation) was conducted on the independent mobility scale and two factors were extracted (Table 2). The three items with high factor loadings (eigenvalues ≥ 0.40) in the first component were going to a shopping center, using public transport to go out, and going to a supermarket or convenience store. Accordingly, they were labeled going out to public places. As the second factor comprised three items, namely, going to a park, going to a friend’s house, and going home from school, the factor was named activities in local settings mainly in the school district. The interfactor correlation was significant and moderately correlated (*r* = 0.30, *p* < 0.001). Subsequently, the mean scale scores of these two factors were calculated and their relationship with other variables was analyzed.

**TABLE 2:** Factor loadings for children’s independent mobility.

	Factor loading	
	
Factors/items	1	2
**Going out to public places**		
Going to a shopping center	0.91	
Public transportation such as a bus or train to go out	0.77	
Going to a supermarket or convenience store	0.57	
**Activities in local settings mainly in the school district**		
Going to a park		0.77
Going to a friend’s house		0.72
Going home from school		0.44

### Relationship Between Independent Mobility and Other Variables

#### Demographic Variables

A three-way analysis of variance was conducted, with school grade (grades 1–6), gender (boy, girl), and birth order (only child, first child with younger siblings, second or subsequent child) as independent variables and the mean scores of the two factors of independent mobility as the dependent variable (Tables 3, 4). There was an interaction between grade and birth order for the first factor of independent mobility, namely, going out to public places [*F*(10,1788) = 1.99, *p* < 0.05, $\eta_{\text{p}}^{2}$ = 0.01]. *Post hoc* tests with Holm correction fixed grades revealed that a simple main effect of birth order only for grade 2 [*F*(2,1788) = 5.64, *p* < 0.01, $\eta_{\text{p}}^{2}$ = 0.01]. The values for the second or subsequent children were lower than for only children and first children with younger siblings (*p*s < 0.05). *Post hoc* tests also revealed simple main effects of grades for only children and second or subsequent children [*F*(5,1788) = 3.00, *p* < 0.05, $\eta_{\text{p}}^{2}$ = 0.01; *F*(5,1788) = 2.41, *p* < 0.05, $\eta_{\text{p}}^{2}$ = 0.01, respectively]. When pair-wise comparisons with Bonferroni correction were made, there was no difference between the grades for the only children. However, for the second or subsequent children, there was a difference between the second and fourth grade (*p* < 0.05). Main effects of grade and gender were also found [*F*(5,1788) = 2.59, *p* < 0.05, $\eta_{\text{p}}^{2}$ = 0.01; *F*(1,1788) = 16.78, *p* < 0.001, $\eta_{\text{p}}^{2}$ = 0.01, respectively]. In relation to grade, Tukey’s *post hoc* comparisons showed a significant difference between grade 1 and 5 (*p* < 0.05). Furthermore, the value for boys was greater than that for girls.

**TABLE 3:** Associations of grade, gender, and birth order with independent mobility.

	Grade											
	
	1						2					
		
	Boy			Girl			Boy			Girl		
				
Independent mobility	Only child	First child with younger sib.	Second or subsequent child	Only child	First child with younger sib.	Second or subsequent child	Only child	First child with younger sib.	Second or subsequent child	Only child	First child with younger sib.	Second or subsequent child
Going out to public places	2.45	2.44	3.40	2.04	2.58	2.33	2.50	2.96	2.38	2.18	2.63	2.00
Activities in local settings mainly in the school district	2.73	2.90	3.00	2.66	2.89	2.00	2.79	3.24	3.23	2.76	3.09	3.25

	**3**						**4**					
		
	**Boy**			**Girl**			**Boy**			**Girl**		
				
**Independent mobility**	**Only child**	**First child with younger sib.**	**Second or subsequent child**	**Only child**	**First child with younger sib.**	**Second or subsequent child**	**Only child**	**First child with younger sib.**	**Second or subsequent child**	**Only child**	**First child with younger sib.**	**Second or subsequent child**

Going out to public places	2.92	2.63	3.08	2.87	2.73	2.11	2.61	2.73	3.10	2.65	2.63	2.77
Activities in local settings mainly in the school district	3.40	3.39	3.43	3.20	3.20	3.30	3.11	3.46	3.65	3.19	3.12	3.41

	**5**						**6**					
		
	**Boy**			**Girl**			**Boy**			**Girl**		
				
**Independent mobility**	**Only child**	**First child with younger sib.**	**Second or subsequent child**	**Only child**	**First child with younger sib.**	**Second or subsequent child**	**Only child**	**First child with younger sib.**	**Second or subsequent child**	**Only child**	**First child with younger sib.**	**Second or subsequent child**

Going out to public places	3.12	2.98	2.95	2.64	2.61	2.72	3.07	2.65	2.92	2.50	2.55	2.65
Activities in local settings mainly in the school district	3.35	3.64	3.69	3.46	3.33	3.50	3.81	3.55	3.59	3.44	3.53	3.59

*Range 1–5.*

**TABLE 4:** Summary of three-way analysis with grade, gender, and birth order.

	Main effects				Interactions	
		
Independent mobility	Grade	Gender	Birth order	Grade × Gender	Grade × Birth order	Gender × Birth order
Going out to public places	1 < 5	G < B	–	–	Grade 2 : S < O, F Second or subsequent : Grade 2 < 4	–
Activities in local settings mainly in the school district	1 < 2, 3, 4, 5, 6 2 < 3, 4, 5, 6 3 < 5, 6 4 < 6	G < B	–	–	Grade 2 : O < S, F Grade 4 : O, F < S Only children : Grade 1 < 3, 4, 5, 6 Grade 2 < 3, 5, 6 Grade 4 < 6 Firstborn with younger sib. : Grade 1 < 2, 3, 4, 5, 6 Grade 2 < 5, 6 Second or subsequent : Grade 1 < 2, 3, 4, 5, 6	–

*B, boy; G, girl; S, second or subsequent child; O, only child; F, firstborn child with younger sibling.*

For the second factor, namely, activities in local settings mainly in the school district, the interaction between grade and birth order was also significant [*F*(10,1788) = 2.11, *p* < 0.05, $\eta_{\text{p}}^{2}$ = 0.01]. *Post hoc* tests with Holm correction fixed grades revealed that the effect of birth order was significant in grades 1, 2, and 4 [grade 1: *F*(2,1788) = 3.27, *p* < 0.05, $\eta_{\text{p}}^{2}$ = 0.00; grade 2: *F*(2,1788) = 8.43, *p* < 0.001, $\eta_{\text{p}}^{2}$ = 0.01; grade 3: *F*(2,1788) = 4.60, *p* < 0.05, $\eta_{\text{p}}^{2}$ = 0.01]. In grade 2, the value for only child was lower and in grade 4, the value for second or subsequent child was greater than the other two (both, *p*s < 0.05). No significant difference was found among birth order for grade 1. *Post hoc* tests with Holm correction fixed birth order revealed that there were simple main effects of grade for all birth orders [only child: *F*(5,1788) = 15.63, *p* < 0.001, $\eta_{\text{p}}^{2}$ = 0.04; first child with younger siblings: *F*(5,1788) = 13.69, *p* < 0.001, $\eta_{\text{p}}^{2}$ = 0.04; second or subsequent children: *F*(5,1788) = 7.55, *p* < 0.001, $\eta_{\text{p}}^{2}$ = 0.02]. The details are presented in Table 4. Furthermore, for the second factor, the main effects of grade and gender were significant [grade: *F*(5,1788) = 28.82, *p* < 0.001, $\eta_{\text{p}}^{2}$ = 0.08; gender: *F*(1,1788) = 14.90, *p* < 0.001, $\eta_{\text{p}}^{2}$ = 0.01]. Tukey’s multiple comparisons revealed that in relation to grade, the value of grade 1 was smaller than all the other grades, the value of grade 2 was smaller than all grades above grade 3, the value of grade 3 was smaller than grades 5 and 6, and the value of grade 4 was smaller than grade 6. Furthermore, the value for boys was greater than that for girls.

#### Area of Residence

A one-way analysis of variance with area of residence (urban, suburban, and rural) as the independent variable revealed that the main effect was not significant for either the first or second factor of independent mobility [*F*(2,1821) = 2.35, 0.99, respectively, both ns, see Table 5].

**TABLE 5:** Associations of area of residence with independent mobility.

	Area of residence		
	
Independent mobility	Urban	Suburban	Rural
Going out to public places	2.63	2.67	2.89
Activities in local settings mainly in the school district	3.28	3.29	3.19
			

#### Residence

We conducted a one-way analysis of variance with the type of dwelling (detached house, low-rise, mid-rise, and high-rise apartment complex) as the independent variable. Those who gave other answers such as duplet apartment and shared house were excluded from the analysis because their specific housing type was not clear. The main effect was not significant for both factor 1 and 2 [*F*(3,1814) = 1.40, 0.79, respectively, both ns, see Table 6].

**TABLE 6:** Associations of residence with independent mobility.

	Residence			
	
		Apartment complex		
		
Independent mobility	Detached house	Low-rise	Middle-rise	High-rise
Going out to public places	2.63	2.67	2.89	2.89
Activities in local settings mainly in the school district	3.28	3.29	3.19	3.19
				

#### Distance From Home to School and Means of Getting to School

A one-way analysis of variance was conducted with distance to school (less than 500 m, between 500 m and 1 km, between 1 and 2 km, and more than 2 km) as the independent variable (Table 7). Although the main effect was not significant for the first factor [*F*(3,1820) = 0.01], it was significant for the second factor [*F*(3,1820) = 6.13, *p* < 0.001, $\eta_{\text{p}}^{2}$ = 0.02). Tukey’s *post hoc* comparisons revealed that the value for less than 500 m was greater than the values for between 1 and 2 km and more than 2 km (both *p*s < 0.01). The values for between 500 m and 1 km were larger than those for between 1 and 2 km and more than 2 km (*p* < 0.05, *p* < 0.01, in descending order).

**TABLE 7:** Associations of distance from home to school with independent mobility.

	Distance from home to school					
	
Independent mobility	Less than 500 m^a^	− 1 km^b^	− 2 km*^c^*	More than 2 km^d^	*Post hoc* test
Going out to public places	2.67	2.67	2.68	2.68	
Activities in local settings mainly in the school district	3.41	3.33	3.20	3.11	a > c, d b > c, d
					

A one-way analysis of variance was also conducted for means of getting to school, specifically accompanied by an adult, school bus, in a group with only children, or alone (Table 8). Although no main effect was found for the first factor [*F*(2,1732) = 1.54], the main effect was significant for the second factor [*F*(2,1732) = 34.57, *p* < 0.001, $\eta_{\text{p}}^{2}$ = 0.04]. Tukey’s *post hoc* comparisons showed that the values for children traveling to school alone were larger than those for the other two groups (traveling to school alone > traveling to school in a group, *p* < 0.05; traveling to school alone > accompanied by an adult or traveling on a school bus, *p* < 0.001). The value of traveling to school in a group was larger than that of traveling to school with an adult or on a school bus (*p* < 0.001).

**TABLE 8:** Associations of means of getting to school with independent mobility.

	Means of getting to school					
	
Independent mobility	Accompanied by an adult, or school bus^a^	Group with only children^b^	Alone^c^	*Post hoc* test
Going out to public places	2.51	2.70	2.70	
Activities in local settings mainly in the school district	2.84	3.29	3.40	a > b > c

#### Neighboring Environment

Exploratory factor analysis using the maximum likelihood method and promax rotation was conducted on the scale of the neighborhood environment. In view of the eigenvalues (≥0.40) and the decay of the scree plot (Table 9), it was deemed appropriate to regard it as a three-factor structure. In relation to the items with factor loadings of 0.40 or higher, the first factor had high values for the following three items: banks, post offices, medical institutions, and public facilities in the neighborhood; shops, supermarkets, and shopping areas within walking distance of the child; and bus stops and train stations within walking distance of the child. Accordingly, the factor was named accessibility to major facilities and places. The second factor had high loadings for high risk of crime, making it unsafe for children to go out, heavy traffic, making it dangerous for children to go out, and graffiti and garbage in some places. The second factor was accordingly named concern about traffic accidents and public security. The third factor had high loadings for several free or inexpensive recreational facilities such as parks, green spaces, playing fields, and public swimming pools, bicycle lanes accessible to bicycles, interesting things such as beautiful scenery, and most roads have sidewalks. The third factor was thus called well-developed infrastructure.

**TABLE 9:** Factor loadings for neighborhood environment.

	Factor loading		
	
Factors/items	1	2	3
**Accessibility to major facilities and places**			
Banks, post offices, medical institutions, and public facilities are in the neighborhood	0.87		
Shops, supermarkets, and shopping areas are within walking distance of the child	0.86		
Bus stops and train stations are within walking distance of the child	0.68		
**Concern about traffic accidents and public security**			
High risk of crime, so it is not safe for children to go out		0.78	
Heavy traffic, so it is dangerous for children to go out		0.54	
Graffiti and garbage in some places		0.42	
**Well-developed infrastructure**			
Several free or inexpensive recreational facilities			0.57
Bicycle lanes that are accessible to bicycles are in the neighborhood			0.51
Interesting things (such as beautiful scenery) are in the neighborhood			0.43
Most roads have sidewalks			0.41

Next, A confirmatory factor analysis was conducted using the maximum likelihood method with the same data in order to assess the factorial validity of the model. The results confirmed the validity of this factor model, as the goodness-of-fit indices for the model were *CFI* = 0.964, *RMSEA* = 0.053, and *GFI* = 0.980.

The correlation coefficients between the mean scores of these three factors and scores of independent mobility were calculated. The results revealed a correlation between the first factor of neighborhood environment and both factors of independent mobility. The first factor of neighborhood environment was negatively correlated (*r* = −0.08, *p* < 0.01) to the first factor of independent mobility and positively correlated (*r* = −0.08, *p* < 0.01) to the second factor (*r* = 0.08, *p* < 0.01). There was also a correlation between the second factor of neighborhood environment and both factors of independent mobility. Both were negatively correlated (*r* = −0.09, *p* < 0.001; *r* = −0.12, *p* < 0.01, respectively). Furthermore, the third factor of neighborhood environment was negatively correlated with the first factor of independent mobility (*r* = −0.06, *p* < 0.05). The correlation coefficients were all low.

#### Using a Bicycle, and Carrying Keys and Cell Phone

A one-way analysis of variance on the association between children’s use of bicycles (cannot ride/does not go out, not often, sometimes, and frequently) and independent mobility confirmed a main effect for the second factor [*F*(3,1820) = 53.90, *p* < 0.001, $\eta_{\text{p}}^{2}$ = 0.08]. Tukey’s *post hoc* comparisons revealed a significant difference among all combinations, with the following results: cannot ride/cannot go out < not often < sometimes < often (cannot ride/cannot go out < not often, *p* < 0.05; all other combinations, *p*s < 0.001). The main effect of the first factor was not significant [*F*(3,1820) = 0.98].

In relation to whether children had an opportunity to go out with a key, a significant difference in both factors of independent mobility was found (*t* = −2.17, *p* < 0.05, $\eta_{\text{p}}^{2}$ = 0.05; *t* = −9.61, *p* < 0.001, $\eta_{\text{p}}^{2}$ = 0.22, respectively). The values were higher when children were allowed to leave the house with a key for both factors.

Subsequently, a one-way analysis of variance was performed to examine the association between having and using a cell phone (not having a cell phone, having a cell phone but never going out with it, and going out with it) and independent mobility. Although there was no main effect for the first factor of independent mobility [*F*(3,1821) = 1.01], that for the second factor was significant [*F*(3,1821) = 16.43, *p* < 0.001, $\eta_{\text{p}}^{2}$ = 0.02]. Furthermore, Tukey’s *post hoc* comparisons revealed that the value for going out with was higher than those of the other two groups (both *p*s < 0.01).

#### Frequency of Lessons

In addition, a one-way analysis of variance was performed on the number of days on weekdays spent attending lessons as the independent variable. The results showed that the main effect was significant for both factors of independent mobility [*F*(3,1820) = 45.43, *p* < 0.001; $\eta_{\text{p}}^{2}$ = 0.07; *F*(3,1820) = 4.66, *p* < 0.01, $\eta_{\text{p}}^{2}$ = 0.01, respectively]. Tukey’s *post hoc* comparisons confirmed that for the first factor, no days had a higher value than the other three groups (all *p*s < 0.001). Furthermore, no days was found to be higher than 1 day and more than 3 days for the second factor (0 days > 1 day, *p* < 0.05; 0 days > more than 3 days, *p* < 0.01).

### Hierarchical Multiple Regression Analysis

In consideration of the purpose of this study, hierarchical multiple regression analysis was conducted to ascertain the impact of each variable on children’s independent mobility comprehensively. Local characteristics and type of residence were excluded from the explanatory variables because the analyses thereof revealed they were not associated with independent mobility. In the analysis, the first step was grade, gender, and birth order, the second was the neighborhood environment, the third was the distance to school and means of getting to school, the fourth was riding a bicycle and going out with a key or cell phone, and the fifth was the number of weekday lessons.

#### Going Out to Highly Public Places

As displayed in Table 10, for the first factor of independent mobility, although the coefficients of determination were not large overall, the increase in values between the fourth and fifth steps was the largest. In the first step, the effect of gender was the largest (β = −0.23), exceeding the effect of grade (β = 0.06). When neighborhood environment was added in the second step, the influence of factor 1 (accessibility to major facilities) and factor 2 (concerns about traffic accidents and public security) were confirmed (β = −0.14 and −0.21, respectively). No influence of new variables was identified in the third and fourth steps. When the number of days of lessons on weekdays was added in the fifth step, while the influence thereof was also confirmed (β = −0.26), the influence of the variables confirmed in the previous steps was maintained.

**TABLE 10:** Results of hierarchical multiple regression analysis predicting the first factor of children’s independent mobility.

	Step 1				Step 2				Step 3				Step 4				Step 5			
									
Variables	*B*	*SE B*	β		*B*	*SE B*	β		*B*	*SE B*	β		*B*	*SE B*	β		*B*	*SE B*	β	
Grade	0.06	0.02	0.08	**	0.06	0.02	0.07	**	0.06	0.02	0.07	**	0.06	0.02	0.07	*	0.06	0.02	0.08	**
Gender	–0.23	0.07	-0.08	**	−0.23	0.07	−0.08	**	−0.23	0.07	−0.08	**	−0.23	0.07	−0.08	**	−0.24	0.07	−0.09	**
Birth order	0.00	0.01	0.01		0.00	0.01	0.00		0.00	0.01	0.00		0.00	0.01	0.00		0.00	0.01	0.00	
Neighborhood environment F1					−0.14	0.05	−0.08	**	−0.15	0.05	−0.08	**	−0.15	0.05	−0.08	**	−0.14	0.05	−0.08	**
Neighborhood environment F2					−0.21	0.06	−0.08	**	−0.21	0.06	−0.08	**	−0.21	0.06	−0.08	**	−0.21	0.06	−0.08	**
Neighborhood environment F3					−0.03	0.06	−0.01		−0.03	0.06	−0.01		−0.02	0.06	−0.01		0.00	0.06	0.00	
Distance to school									0.00	0.04	0.00		0.00	0.04	0.00		−0.02	0.04	−0.01	
Means of getting to school									0.07	0.05	0.03		0.06	0.05	0.03		0.07	0.05	0.03	
Bicycle													−0.02	0.03	−0.02		−0.01	0.03	−0.01	
House key													0.12	0.08	0.04		0.14	0.07	0.05	
Cell phone													−0.02	0.04	−0.01		0.01	0.04	0.00	
Frequency of lesson/cram school																	−0.26	0.03	−0.22	**
*R* ^2^	0.01				0.03				0.03				0.03				0.08			
Change in *R*^2^						0.01				0.00				0.00				0.05		
*F* for change in *R*^2^		7.85	**			8.24	**			0.81				0.88				87.24	**	

**p < 0.01,*p < 0.05.

#### Activities in Local Settings, Mainly Within the School District

In relation to the second factor of independent mobility (Table 11), in the first step, the effects of grade, birth order, and gender were all significant, with gender having the largest effect (β = −0.15). As a result of adding the neighborhood environment in the second step, the influence of the first factor (accessibility to major facilities) and the second factor (concerns about public security and traffic) were confirmed (β = 0.07, −0.13, respectively). Moreover, the influence of the second factor of neighborhood environment was maintained until the fifth step. In the third step, the influence of distance to school and means of going to school were confirmed (β = −0.05, 0.16, respectively). The influence of these two variables was maintained until the fifth step. In the fourth step, the effects of going out by bicycle and going out with a key were confirmed (β = 0.11, 0.12, respectively). Finally, in the fifth step, the influence of learning was observed (β = −0.10).

**TABLE 11:** Results of hierarchical multiple regression analysis predicting the second factor of children’s independent mobility.

	Step 1				Step 2				Step 3				Step 4				Step 5			
									
Variables	*B*	*SE B*	β		*B*	*SE B*	β		*B*	*SE B*	β		*B*	*SE B*	β		B	SE B	β	
Grade	0.14	0.01	0.29	^**^	0.14	0.01	0.28	^**^	0.13	0.01	0.27	^**^	0.09	0.01	0.19	^**^	0.10	0.01	0.20	^**^
Gender	–0.15	0.04	−0.09	^**^	−0.15	0.04	−0.09	^**^	−0.15	0.04	−0.09	^**^	−0.12	0.04	−0.07	^**^	−0.13	0.04	−0.08	^**^
Birth order	0.01	0.00	0.06	^**^	0.01	0.00	0.06	^**^	0.01	0.00	0.05	*	0.01	0.00	0.05	*	0.01	0.00	0.05	*
Neighborhood environment F1					0.07	0.03	0.06	*	0.03	0.03	0.03		0.03	0.03	0.03		0.03	0.03	0.03	
Neighborhood environment F2					−0.13	0.04	−0.08	^**^	−0.12	0.03	−0.08	^**^	−0.11	0.03	−0.07	^**^	−0.12	0.03	−0.08	^**^
Neighborhood environment F3					0.02	0.03	0.02		0.02	0.03	0.02		0.00	0.03	0.00		0.01	0.03	0.01	
Distance to school									−0.05	0.02	−0.06	^**^	−0.05	0.02	−0.05	*	−0.06	0.02	−0.06	^**^
Means of getting to school									0.16	0.03	0.12	^**^	0.15	0.03	0.11	^**^	0.15	0.03	0.12	^**^
Bicycle													0.11	0.02	0.15	^**^	0.11	0.02	0.16	**
House key													0.12	0.04	0.07	^**^	0.13	0.04	0.08	^**^
Cell phone													−0.02	0.02	−0.02		−0.01	0.02	−0.01	
Frequency of lesson/cram school																	−0.09	0.02	−0.14	^**^
*R* ^2^	0.11				0.12				0.14				0.16				0.181			
Change in *R*^2^						0.01				0.02				0.02				0.018		
*F* for change in *R*^2^		68.64	^**^			7.53	^**^			21.04	^**^			16.61	^**^			37.348	^**^	

**p < 0.01, *p < 0.05.

## Discussion

After starting elementary school, Japanese children have increasing opportunities to be with their friends after school and even go out alone sometimes. However, in Western countries and many other developed countries, parents tend to be concerned about their children being involved in traffic accidents and crimes and thus, may be cautious about their children going out alone. Parents in Western countries commonly drive their children to and from school as well as extracurricular activities. Some researchers have derisively referred to this parental behavior as chauffeuring (Nordbakke, 2019). This decrease in independent mobility has been prevalent since the 1980s and its adverse effects on physical, cognitive, and psychosocial development have been highlighted. In this study, we investigated the development of independent mobility and its related factors in elementary school children in Japan. In addition to the variables that have been identified in previous studies, this study examined the frequency of attending after-school extracurricular activities (naraigoto) on weekdays.

### Independent Mobility

With regard to independent mobility, while items related to going out to supermarkets, malls, and using trains/buses were classified as the first factor, those related to going out to parks, visiting friends’ houses, and traveling alone from school were grouped together as the second factor. The former may be regarded as going out alone with children to public places and/or those that are inaccessible without public transportation. Because of the many risks of encountering unspecified, unknown strangers, parents tend to be cautious about allowing their children to access these places alone. On the contrary, the range of activities for the second factor was mainly limited to the school district, with which the children are familiar. Furthermore, those they meet are likely to be friends and acquaintances. Consequently, parents are likely to be relatively tolerant of their children going to such places. Although there was a moderate correlation between the two factors, because they were separate factors suggests parents perceive these as qualitatively differentiated places.

### Relationship Between Independent Mobility and Other Variables

#### Demographic Variables

There was an association between grade and gender for both factors of independent mobility. Furthermore, independent mobility generally increased the higher the grade and was lower for girls than for boys. This concurs with previous studies that have found lower independent mobility among girls (Hillman et al., 1990; O’Brien et al., 2000; Johansson, 2006; Brown et al., 2008; Fyhri and Hjorthol, 2009). There was also a significant interaction between school grade and birth order. Independent mobility was lower for second or subsequent children in grade 2 in relation to going to public places. However, with respect to going out in the school district, independent mobility was lower for only children in grade 2 and higher for second or subsequent children in grade 4. Previous studies throughout the world have consistently revealed that second and subsequent children enjoy enhanced independent mobility (Canada: Cervesato and Waygood, 2019; Australia: Christian et al., 2016). This study differs from previous studies in that the analysis was performed by classifying the data into public places and school districts, which may be a way of classifying Japan’s unique local environment. Because previous studies have not examined the interaction between school grade and birth order, further comparisons with other countries are imperative to examine these issues.

#### Environment-Related Variables

The parents were more willing to allow their children to engage in activities on their own within the school district when the school was located close to their home and/or when their children went to school on their own. Children whose schools are located close to their homes generally travel to school alone, which may suggest that their parents are relatively accustomed to their children going out alone and more likely to tolerate their children going out by themselves. On the contrary, the distance from home to school and the means of traveling to school were not related to going out to highly public places and opportunities of meeting unknown people. As noted previously, parents may perceive familiar local places and other public places differently. Furthermore, even if children are accustomed to going to school alone and/or engaging in activities alone in the school district, it does not mean that they will be allowed to go to more public places. It may be speculated that there is a considerable psychological barrier between these two types of independent mobility.

Three factors were identified in relation to the family’s neighborhood: accessibility to major facilities and places, concern about traffic accidents and public security, and well-developed infrastructure. First, the more concerned parents were about traffic accidents and public security, the less likely their children went out, both within the school district and to highly public places. Studies have revealed that concern about traffic accidents result in children’s low independent mobility (e.g., United States: Janssen et al., 2016; Italy: Prezza et al., 2005). Furthermore, only a scarcity of Japanese has shown that concern about traffic accidents affects children’s low independent mobility (Drianda and Kinoshita, 2011). While neighborhood security influences children’s independent mobility (United States: Janssen et al., 2016; New Zealand: Oliver et al., 2011), the results of this study revealed a parallel tendency in that high volumes of traffic as well as concerns about crime are likely to undermine children’s independent mobility. On the contrary, it was revealed that children whose neighborhoods were well organized were less likely to be allowed to visit public places alone. These neighborhoods were typically characterized by a well-organized suburban environment, with safe spaces for children to play freely. Children may not be motivated to go out beyond these areas. Nevertheless, at some point in their development, children should be able to travel by train or bus and go to various public places such as commercial facilities, without being accompanied by an adult. In this sense, surroundings that are over-protected may limit children’s self-support and independent activities. Accordingly, it is imperative that adults monitor when and how to encourage children’s independent activities. Nevertheless, it is noteworthy that the correlation coefficients between neighborhood environment and independent mobility were modest, with even the highest values exceeding 0.1 slightly.

#### Using Bicycle, and Carrying Keys and Cell Phone

In relation to going out unaccompanied by adults on bicycles and going out with keys and cell phones, the second factor of independent mobility was found to be positively associated with activities in the school district. Similarly, data from Australia have confirmed that male children’s independent mobility is positively affected by bicycle use. Malone and Rudner (2011) found that children’s bicycle use is relatively acceptable in Japan. Bicycles allow one to travel faster and farther away from home, even though traffic accidents may be cause for concern. Although the use of bicycles may be limited to the school district, they may reduce the restrictions parents place on their children going out alone. Children who have a key for their home are often referred to as latchkey kids (Pulkkinen, 2004). While some perceive latchkey kids to be from impoverished families, especially those where both parents work, others view children’s ownership of a key as an indicator of their ability to be self-reliant after school (Rajalakshmi and Thanasekaran, 2015). Japan is a relatively safe country. Furthermore, they enjoy higher tolerance for children to unlock the door and go out by themselves when their parents are not home than in other countries. Moreover, key control may signify parental confidence in children (Ayllón et al., 2019), thus ensuring their independent mobility. Children’s possession of keys in the school district as well as other places is indicative of their parents’ trust in them. While some studies have shown going home without a cell phone is not related to children’s independent mobility (Shaw et al., 2015), others have demonstrated a positive effect thereof (Chaudhurya et al., 2019). When children have cell phones, parents are able to contact their children, thus reducing obstacles related to letting them go out unaccompanied by an adult (Fyhri et al., 2011). There are services in Japan that employ cell phones to check the children’s locations on a map. Moreover, parents increasingly allow their children to have cell phones at earlier ages (Ministry of Health Labour and Welfare, 2021). Although there is insufficient data on how cell phones are actually used in families with children, it is highly likely that parents are willing to allow their children to move around freely because they are comfortable with the idea that they can always check on their whereabouts and contact them. However, it is noteworthy that the association was limited to activities within the school district and not related to children’s outings to public places. Although visiting shopping centers, supermarkets, and using trains and buses may afford children more opportunities to leave home, parents may be unable to get a signal or accurately identify the location, thus making parents more cautious about their children’s activities outside the school district.

#### Frequency of Lessons

Data from Western countries have revealed participation in organized activities has been increasing in parallel with the decline in children’s independent mobility (Fyhri et al., 2011). Furthermore, participation in such activities is more meaningful for children’s development than simply playing outdoors purposelessly. Recently, children’s play activities have been conducted in a safe and secure setting set up and supervised by adults, with a definite number of members gathering at a definite time. However, Holloway and Pimlott-Wilson (2014) cautioned against institutionalizing children’s play in this way. Zeiher (2001) noted critically that children are passively transported from one place to another in a car driven by their parents, compared the places where organized activities are conducted to islands, and added that children’s lives have become insularized. After-school extracurricular activities are also popular in Japan, with approximately 30% of 3-year-olds and almost half of 4-year-olds attending lessons of some kind. As children progress through grades, participation in cram schools that specialize in academics have gradually started to replace lessons (Benesse Institute for Educational Research, 2016). Omiya et al. (2011) noted that more than 30% of fourth- to sixth-graders attended lessons or cram school at least 3 days a week. This concurs with this study’s findings that 25.3% of the children attended some sort of lessons or cram school at least 3 days a week. The current data are limited to weekdays, thus suggesting the possibility that this figure would be higher if weekends were included. The analysis revealed that children who did not attend lessons were more likely to be allowed to go to familiar places in the school district as well as to public places than their counterparts who attended the lessons. Furthermore, children who participate more in organized activities are less likely to participate in free play (Engelen et al., 2015). Japanese data have also indicated that children who attend lessons or cram schools frequently tend to spend more time playing games indoors with their friends than playing outside (Omiya et al., 2011). Although organized activities such as extracurricular lessons and cram schools may have a positive effect on children’s development in relation to acquiring knowledge and skills, such activities also reduce the amount of time children are able to spend on their own outdoors, which may have a negative impact on their development from multiple perspectives.

### Hierarchical Multiple Regression Analysis

Hierarchical multiple regression analyses were conducted to determine whether the number of lesson days influenced independent mobility, even after the other variables were considered. The results revealed although the explanatory power of the model was significant for both components of independent mobility, the fit of the model was slightly better for activities within the school district with which the children were familiar. As expected, the effect of school grade as a demographic variable remained until the final step. However, the partial regression coefficient revealed that gender had an even greater influence, which was stronger in public places. Other than the demographic variables, concerns about neighborhood security and traffic were revealed to be restrictive factors for the two different types of independent mobility. These findings indicate that gender and neighborhood security/traffic conditions affect independent mobility, which concurs with research in other countries (Prezza et al., 2005; Johansson, 2006; Janssen et al., 2016). On the contrary, travel from home to well-known places had a negative effect on independent mobility to more public places. Regarding this association, future studies will need to confirm the underlying evidence, for example, by conducting individual interviews.

In relation to independent activities at locations within the school district, the proximity of their homes to school and going to school on their own had positive effects. It is thought that the closer the distance between the home and school, the more likely it is that children will attend school alone. In such instances, it can be that parents are not afraid of their children going out alone, which may be the reason they are willing to allow their children to leave after school alone within the school district. Again, reinforcing evidence through interviews will be necessary in the future.

Furthermore, being permitted to use bicycles and leave the house with a key also promoted independent mobility in the school district. While it is common for children to travel to school by bicycle in some countries (Tranter and Whitelegg, 1994; O’Brien et al., 2000; Fyhri et al., 2011), this is not common in Japan. However, studies have indicated that Japan is relatively tolerant of children who ride their bicycles home after school (Malone and Rudner, 2011). However, the findings of this study revealed that such parental tolerance was limited to activities within the school district and that they may have been cautious about their children’s activities beyond the school district. A limited number of studies have revealed that children travel to and from school by themselves when they are given keys (New Zealand: Ayllón et al., 2019). It may be assumed that when children are given a key, there will be no one at home when they come home from school. In Japan, while the children are young, many employed mothers leave work early and return home before their children come home from school. If there is no one at home when children return, one may assume parents are not involved in the same activities at their children. Although this may indicate these children are more independent of their parents, this is limited to their independent mobility within the school district.

It is noteworthy that after controlling for other variables, the impact of the number of lesson days on children’s independent mobility remained significant. Furthermore, the partial regression coefficients revealed that this impact was particularly powerful for independent mobility to public places. In many countries, parents are often expected to drop off and pick up their children at an organized activity, but in Japan, children may go to lessons by themselves (Shimada et al., 2010; Omiya et al., 2011). Unfortunately, in this study, we did not obtain data on whether parents transported their children to and from their lessons or whether the children traveled by themselves. Regardless of how they travel to and from lessons, those who attend lessons do not have as much free time as those who do not attend lessons. Omiya et al. (2011) found that children who take lessons tend to spend their limited free time indoors. Furthermore, if children spend a limited amount of free time outside due to the increased frequency of lessons, parents may be discouraged from allowing their children to go outside unaccompanied by an adult. It is recommended that future studies examine whether parents take their children to lessons or whether they go alone (or with friends) and explore why frequent attendance at lessons leads to a decrease in children’s independent mobility.

## Conclusion and Future Direction

This study examined the development of children’s independent mobility, which is characterized by going out without an adult as well as the factors associated with such among Japanese mothers with elementary school-aged children. In addition to the demographic variables, residential area, neighborhood environment, and the availability of devices such as bicycles, household keys, and cell phones, which have been revealed to be related to independent mobility in previous studies, the present study specifically explored the effects of activities that constitute a large part of children’s current after-school activities, including lessons and cram schools. Independent mobility was classified as activities in public places, where encounters with strangers are more frequent, and those within the school district, which are more familiar to children. Hierarchical multiple regression analysis revealed that independent mobility increased as the children advanced through the grades in both contexts. Furthermore, boys were more actively involved in independent activities than girls. Moreover, concerns about traffic accidents and crime influenced the lower level of independent mobility in both contexts. In addition, the availability of stores, banks, and public transportation for accessing distant places restricted children’s exposure to public places. In addition, proximity to school from home, use of bicycles, and permission to carry a key had a positive effect on independent activities within the school district.

Possibly, the most intriguing finding was that the more days the children spent attending lessons or cram school, the more likely this would have a negative impact on independent mobility in both settings, with a particularly strong effect on activities in public places. While lessons and cram schools may be effective in promoting children’s educational knowledge and technical skills, they may also thwart their opportunities to engage in unstructured activities and thus, hinder their physical, cognitive, and social development. However, further research is recommended in Japan as well as other countries throughout the world.

## Data Availability Statement

The raw data supporting the conclusions of this article will be made available by the author, without undue reservation, to any qualified researcher.

## Ethics Statement

The studies involving human participants were reviewed and approved by Chukyo University Ethical Review Committee. Written informed consent for participation was not required for this study in accordance with the national legislation and the institutional requirements.

## Author Contributions

The author confirms being the sole contributor of this work and has approved it for publication.

## Conflict of Interest

The author declares that the research was conducted in the absence of any commercial or financial relationships that could be construed as a potential conflict of interest.

## Publisher’s Note

All claims expressed in this article are solely those of the authors and do not necessarily represent those of their affiliated organizations, or those of the publisher, the editors and the reviewers. Any product that may be evaluated in this article, or claim that may be made by its manufacturer, is not guaranteed or endorsed by the publisher.
